# Comparative genomics of the monophasic variant of *Salmonella* Typhimurium: analysis of Colombian genomes and their relationship with international lineages

**DOI:** 10.1128/spectrum.03870-25

**Published:** 2026-09-17

**Authors:** Paloma Cuenca-Arias, Jeisson Alejandro Triana Diaz, Diego Andrés Prada-Cardozo, Efraín Montilla-Escudero, Héctor Alejandro Ruiz-Moreno, Lucy Angeline Montaño, Edna Catering Rodríguez, José Miguel Villarreal Ascencio, Iliana Chamorro-Tovar, Irina Barrientos, Adriana Pulido-Villamarín, Ana Karina Carrascal-Camacho, Magdalena Wiesner

**Affiliations:** 1 Grupo de Microbiología, Instituto Nacional de Salud 67626, Bogotá, Colombia; 2 Grupo de Genómica de Microorganismos Emergentes, Dirección de Investigación en Salud Pública, Instituto Nacional de Salud, Bogotá, Colombia; 3 Grupo de Investigación en Enfermedades Infecciosas, Universidad Nacional de Colombia 28021, Bogotá, Colombia; 4 Grupo de Bioquímica y Biología Molecular de las Micobacterias, Universidad Nacional de Colombia 28021, Bogotá, Colombia; 5 Grupo de Biotecnología Ambiental e Industrial-GBAI, Food Safety Research Hotbed, Emerging Microorganisms Line, Department of Microbiology, Faculty of Sciences, Pontificia Universidad Javeriana 27964, Bogotá, Colombia; 6 Agricultural Research Unit UNIDIA, Line of Epidemiology and Animal Health, Department of Microbiology, Faculty of Sciences, Pontificia Universidad Javeriana 27964, Bogotá, Colombia; 7 Grupo de investigación en Ciencias Biológicas y Químicas, Facultad de Ciencias, Universidad Antonio Nariño, Bogotá, Colombia; Virginia Polytechnic Institute and State University, Blacksburg, Virginia, USA

**Keywords:** monophasic variant *Salmonella *Typhimurium, antimicrobial resistance, genomic surveillance, One Health

## Abstract

The monophasic variant of *Salmonella enterica* serovar Typhimurium (STVM) represents a growing threat to global public health owing to its wide dissemination, capacity to adapt to multiple hosts, and antimicrobial resistance. In this study, 98 STVM isolates recovered in Colombia (57 from humans and 41 from pig farms and abattoirs) were genomically characterized between 2015 and 2022 and compared with 102 representative genomes of international lineages by whole-genome sequencing (WGS) and phylogenomic analysis. Phylogenomic analysis revealed the existence of two well-defined endemic lineages in Colombia (Clusters 1 and 2), arising from independent introduction events and subsequent local stabilization. Both lineages comprise isolates of human and swine origin without clear phylogenetic separation by host species, suggesting active zoonotic cocirculation and closely integrated interspecies transmission dynamics. Marked differences were observed in the accessory genome, including the differential presence of prophages (e.g., Gifsy-2, Fels-2, SW9), virulence plasmids, and resistance profiles. The Colombian lineages exhibited a high frequency of the pSTV plasmid (85%, *n* = 84/98) and a substantial burden of resistance determinants to quinolones (such as *qnrB19*, 74.5%; *gyrA* S83F mutation, 19.4%), phenicols (*floR*), tetracyclines (*tetA*, *tetB*), β-lactams (*bla*TEM-1B), and heavy metals. In contrast, the Colombian genomes clustered with the European ST34 lineage lacked pSTV but retained resistance and heavy metal operons. These findings demonstrate that international and endemic lineages coexist in Colombia with independent evolutionary trajectories, underscoring the need to strengthen genomic surveillance under the “One Health” approach to anticipate emerging threats and develop integrated control strategies.

**IMPORTANCE:**

The monophasic variant of *Salmonella* Typhimurium (STVM) has emerged as a predominant serovar in both humans and swine internationally. In Colombia, a fundamental question driving this study was whether local isolates belonged to international lineages or represented endemic strains. This study provides the first comprehensive genomic characterization demonstrating that two Colombian endemic lineages circulate simultaneously between humans and pigs without phylogenetic separation by host species, confirming active zoonotic transmission. The results demonstrate the coexistence of both lineages, each with distinctive repertoires of mobile genetic elements and specific antimicrobial resistance profiles. Understanding these transmission dynamics and evolutionary patterns is crucial for public health, as it demonstrates how zoonotic pathogens can establish locally adapted lineages with distinct resistance patterns. The genomic evidence of sustained interspecies circulation highlights the critical need for integrated surveillance strategies under the “One Health” framework. This will enable anticipating emerging threats, tracing transmission routes, and developing targeted interventions in food production systems.

## INTRODUCTION

Salmonellosis is among the most common foodborne illnesses worldwide, with an estimated 95.1 million cases of acute gastroenteritis per year (1). The etiological agent is the bacterium of the genus *Salmonella*, which is classified into more than 2,600 serovars that affect both humans and animals and strongly affect public health, livestock production and the economy (2). Among the most relevant serovars is *Salmonella enterica* subspecies *enterica* serovar Typhimurium (*S*. Typhimurium), one of the leading causes of human salmonellosis worldwide.

In recent decades, a monophasic variant of *S*. Typhimurium (STVM) has emerged, characterized by the loss of the second flagellar phase mediated by the *fljAB* operon. Initially reported in the late 1990s in Europe and the United States, this variant has progressively replaced *S*. Typhimurium in both human clinical cases and the swine production chain, increasing the risk of zoonotic transmission (3–5).

Genomic analyses of STVM have revealed the loss of chromosomal regions, the acquisition of antibiotic and heavy metal resistance genes, and variations in multidrug resistance (MDR) profiles and phage types. These characteristics suggest either multiple independent emergence events or a local origin followed by global dissemination, giving rise to distinct clonal lineages (6, 7). Among the most recognized are the European ST34 lineage, the Spanish ST19 lineage, and the USA ST19 lineage. The first two are multiresistant, lack the *fljAB* operon and the virulence plasmid pSLT, and have acquired the SGI-4 genomic island. In contrast, the USA ST19 lineage retains part of the *fljAB* operon and is generally susceptible to antimicrobial agents (8, 9).

In Colombia, STVM has become a predominant serovar in both humans and the swine production chain. In humans, the first report was documented in 2015 through the Acute Diarrhea Disease laboratory surveillance system. By 2024, 1,126 isolates serotyped as monophasic had been identified, of which 280 (24.86%) were confirmed by PCR as STVM, placing it fourth among the most frequently identified serotypes (10–13). In the swine sector, recent studies report a high prevalence, with detection in 41.2% of pig farms and in 64% of samples from pig abattoirs (14) (I. Barrientos, A. K. Carrascal, A. Pulido-Villamarín, I. Chamorro, L. C. Veloza, M. Wiesner, F. Rojas, M. Pérez, J. E. Olsen, Y. Hounmanou, A. Dalsgaard, L. Alban, unpublished data). These findings suggest possible zoonotic transmission, which has not yet been explored at the genomic level.

Previous studies based on PCR and genomic analysis of Colombian human clinical isolates have revealed the presence of four main lineages in the country: the European ST34 lineage, the USA ST19 lineage, and two local lineages exhibiting distinctive genetic characteristics relative to international lineages (15, 16). In particular, Li et al. (16), identified two multiresistant STVM clusters, (including Cluster 7, linked to the reference strain LT2), through the analysis of 270 isolates of *S*. Typhimurium obtained from blood cultures in Colombian patients between 1997 and 2017. Within this cluster, they proposed that subcluster 7.3, originated from Colombia, was characterized by the presence of the resistance genes *qnrB19* and *floR*. However, whether the STVM isolates recovered from the Colombian pig farms and abattoirs are genetically related to those from humans — and whether both groups share the same lineages or exhibit evolutionary divergence — remains unknown. The objective of this study was therefore to analyze the genetic relationship between STVM isolates recovered from humans and from pig farms and abattoirs in Colombia, and to reconstruct the evolutionary history of these isolates through comparative phylogenomic analysis of 98 Colombian genomes against a set of 102 international reference genomes.

## MATERIALS AND METHODS

### Colombian clinical genomes of STVM

In this study, 57 human clinical genomes of monophasic variant of *S*. Typhimurium (STVM) recovered from feces and blood cultures between 2015 and 2022 in Colombia were analyzed (Table S1—Metadata). The isolates were serotyped by the Kauffman–White–Le Minor scheme (17) and confirmed by PCR as the monophasic variant of *S*. Typhimurium STVM (18). Antimicrobial susceptibility was evaluated for ampicillin, chloramphenicol, streptomycin, tetracycline, gentamicin, amikacin, nalidixic acid, trimethoprim, ciprofloxacin, ceftazidime, and cefotaxime by the disk diffusion method (Kirby-Bauer) in accordance with the recommendations of the 32nd edition (M100-Ed32) of the CLSI 2022 (19).

Genomic DNA was extracted using the PureLink Genomic DNA Mini Kit (Invitrogen, USA). Genomic libraries were prepared with a Nextera XT kit (Illumina, USA), and sequencing was performed on a MiSeq system (Illumina, USA). Reads were quality-filtered with Trimmomatic v0.39 (https://github.com/timflutre/trimmomatic) and assembled with SPAdes v3.14.0 (https://github.com/ablab/spades) using default parameters. Serotypes were confirmed *in silico* using SeqSero2 (https://github.com/denglab/SeqSero2).

### Colombian pig farms and abattoirs STVM

Forty-one STVM sequences previously obtained from pig farms and abattoirs in Colombia (14) (I. Barrientos, A. K. Carrascal, A. Pulido-Villamarín, I. Chamorro, L. C. Veloza, M. Wiesner, F. Rojas, M. Pérez, J. E. Olsen, Y. Hounmanou, A. Dalsgaard, L. Alban, unpublished data) were selected for analysis (Table S1—Metadata).

### Reference genomes

In all analyses, the STVM lineage reference strains of Spanish origin ST19 (accession number: DRR106818), European ST34 (accession number: ERR985368), and USA ST19 (accession number: ABAO01000000) were used together with the genome of *S*. Typhimurium LT2 (accession number: AE006468) (20–23).

### Selection of international STVM genomes

A systematic search was conducted in the EMBL-EBI and PubMed databases using the keywords “phylogeny” and “monophasic” *Salmonella*. Selection criteria included the terms “Typhimurium,” “clonal lineage,” “Spanish lineage,” “USA lineage,” “European lineage,” and geographic location, year of collection, and source (human, food, or animal). A total of 102 genomes were selected from diverse sources (animal, environmental, and human clinical) and countries: Canada (*n* = 38), the United States (*n* = 18), Denmark (*n* = 13), Australia (*n* = 7), Japan (*n* = 7), Portugal (*n* = 7), Brazil (*n* = 3), New Zealand (*n* = 3), Spain (*n* = 3), the United Kingdom (*n* = 1), Ireland (*n* = 1), and Malaysia (*n* = 1). International genomes were selected to represent characterized lineages without clone removal, as our objective was to compare Colombian isolates against established international lineage representatives (see Table S1—Metadata).

### Typing

Sequence types (STs) were determined using the 7-gene MLST scheme implemented in MLST tool v2.0, available on the Center for Genomic Epidemiology platform (https://www.genomicepidemiology.org/).

Deletion patterns in the *fljAB-hin* region were determined through comparative analysis of assembled genomes against the reference strain *S*. Typhimurium LT2. Although read mapping to confirm deletions was not performed, the consistency of deletion patterns within clusters and their concordance with previously characterized reference strains support the robustness of our findings.

Plasmid incompatibility groups were identified with PlasmidFinder (24). The resistome was characterized with ABRicate available on the Galaxy web platform version 1.0.1 (https://usegalaxy.org/), which incorporates reference databases of antimicrobial resistance genes including ARG-ANNOT, CARD, NCBI AMRFinderPlus, and ResFinder. The virulome was assessed using the VFDB (25).

Prophage regions were determined using the PHASTEST tool (available at http://phastest.ca), which classifies prophage regions as complete, questionable, or incomplete according to a predetermined algorithmic configuration; sequences with identities greater than 90% were retained (26).

Genomes were annotated using Prokka v1.14.6, and the resulting GFF3 files were used as input for pangenome analysis with ROARY V3.13.0 (available in Galaxy, https://usegalaxy.org/) (27). Results were visualized using the PHANDANGO program (28).

### Phylogenomic analysis

Phylogenomic trees were constructed based on SNP (single nucleotide polymorphism) analysis using CSIPhylogeny from the web platform of the Center for Genomic Epidemiology (http://www.genomicepidemiology.org/) (29) under the following default conditions (https://www.genomicepidemiology.org/): minimum depth at SNP positions: 10; relative depth at SNP positions: 10; minimum distance between SNPs: 10; minimum SNP quality: 30; minimum read mapping quality: 25; minimum Z score: 1.96. The *S*. Typhimurium LT2 genome was used as the reference. The maximum-likelihood phylogeny was inferred from the alignment of pseudogenomes derived from variant positions, and both the phylogenetic tree and Table S1—Metadata were visualized with Microreact (30). To assess whether Colombian isolates were related to internationally reported lineages, their phylogenomic profiles were compared with representative strains previously characterized in global studies. The analysis evaluated cluster membership in recognized clonal lineages, including internationally endemic ones, and explored whether the Colombian isolates grouped with global lineages or represented a distinct Colombian lineage.

To evaluate the robustness of the phylogenomic clusters identified above within a broader global context, an independent expanded data set was constructed through a systematic search in EnteroBase (https://enterobase.warwick.ac.uk) (31), querying all available *Salmonella enterica* assemblies annotated with antigenic formulas corresponding to the monophasic variant of *S*. Typhimurium in all recognized notation variants (4,[5],12:i:-; 1,4,[5],12:i:-; 4,5,12:i:-; I 4,[5],12:i:-; and equivalent representations), retrieving approximately 22,000 genomes. A stratified sampling strategy was applied using continent as the primary stratum and country as the secondary stratum. For countries with 30 or more available genomes, up to 16 representative assemblies were selected; for countries with fewer than 30 available genomes, all assemblies passing quality criteria were retained. Proportionally larger quotas were applied for the three most represented countries in the database: United Kingdom (*n* = 155), United States (*n* = 69), and Canada (*n* = 69). Genomes were included if they met the following quality thresholds: minimum coverage ≥18×, N50 ≥ 20,000 bp, and assembly length consistent with *Salmonella enterica* (4.8–5.2 Mb). After quality filtering, 507 international genomes from 22 countries across six continents were retained: Europe (*n* = 268), North America (*n* = 145), Asia (*n* = 52), South America (*n* = 19), Oceania (*n* = 16), and Africa (*n* = 6), spanning collection years 2000–2026 and including human (*n* = 274), livestock (*n* = 100), poultry (*n* = 39), environmental (*n* = 26), wildlife/other (*n* = 5), and unspecified/not determined (*n* = 63) sources. These 507 international genomes were combined with the 95 Colombian isolates described above, yielding a final data set of 603 genomes (including the *S*. Typhimurium LT2 reference genome, NC_003197.2, after removal of duplicates and genomes not meeting quality thresholds. The expanded phylogenomic tree is presented as Fig. S2 and is interactively available at https://microreact.org/project/5zJKTrm94gQTkDGiMvGoQp-newenterobasetree (See Table S1—Metadata)

### Pairwise SNP distance analysis from the expanded data set (603 genomes)

Pairwise core SNP distances were calculated from the extended whole-genome SNP alignment generated by CSIPhylogeny using snp-dists v0.8.2 (29, 32). This tool produces a symmetrical *N* × N matrix reporting the number of core SNP differences between each pair of isolates in the alignment. Distances were analyzed separately for each endemic Colombian cluster (Cluster 1, *n* = 56 isolates; Cluster 2, *n* = 20 isolates) and for Cluster 2 isolates vs the closest international genomes identified in the expanded EnteroBase data set (Fig. S2). Pairwise distances were interpreted according to established epidemiological thresholds for *Salmonella* WGS-based surveillance: <10 SNPs as indicative of closely related isolates consistent with outbreak-level transmission (33); 10–25 SNPs as consistent with prolonged transmission (>1 year) (34); and ≤38 SNPs as the boundary of the pandemic clonal expansion for ST34 lineages (35). Results are presented in Table S2 and are integrated into the phylogenomic interpretation provided in the Results and Discussion sections.

**Fig 1:**
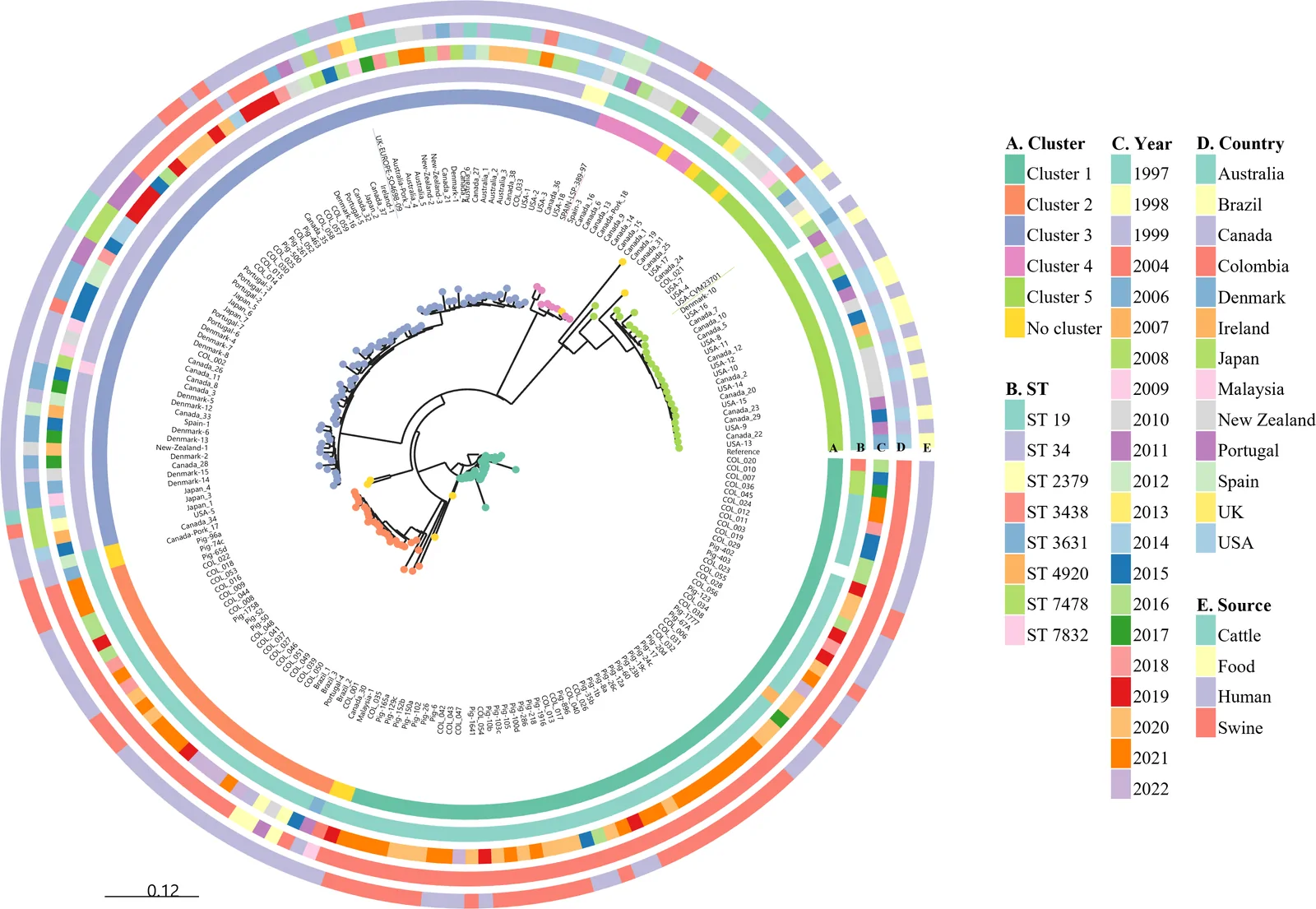
Phylogenetic analysis of Colombian and international STVM genomes. Maximum-likelihood phylogenetic tree inferred from SNP variants. STVM isolates collected between 1997 and 2022 from 13 countries are grouped into five distinct clusters. Leaf node colors indicate cluster assignment: turquoise (Cluster 1, Colombian endemic), salmon (Cluster 2, Colombian endemic), purple (Cluster 3, European ST34), pink (Cluster 4, Spanish ST19), and green (Cluster 5, USA ST19); genomes not assigned to any cluster are shown in light yellow. The color strips to the right of the tree display associated metadata, read from the innermost to the outermost ring: *Cluster*, *ST*, *Year*, *Country*, and *Source*, as detailed in the adjacent legend. Reference strains representative of international lineages are labeled directly on the tree: UK-EUROPE-SO4698-09 (European ST34), SPAIN-LSP-389-97 (Spanish ST19), and USA-CVM23701 (USA ST19).

Phylogenetic clusters were defined based on three criteria applied consistently across both the primary (200-genome) and expanded (603-genome) data sets: (i) phylogenetic topology with bootstrap support ≥70%, (ii) concordance with sequence type (ST) composition, and (iii) consistency with previously characterized international lineages (European ST34, Spanish ST19, and USA ST19). Genomes that did not fulfill these three criteria simultaneously were classified as unassigned. Specifically, two isolates from the United States (US17) and Canada (Canada_1), both assigned ST2379, did not cluster with any of the five defined groups based on their distinct branch length and phylogenetic topology; they are therefore shown as unassigned in Fig. 1.

### Bayesian analysis—analysis of time in phylogeny

To elucidate the timing of introduction of the monophasic variant of *S*. Typhimurium in Colombia, a Bayesian phylodynamic analysis was performed. An initial maximum-likelihood phylogeny was constructed using IQ-TREE v1.6.12 to establish tree topology and select the optimal substitution model (GTR + F + ASC + G4) by Bayesian Information Criterion (BIC) (36). This topology was then used as the basis for Bayesian time-calibrated analysis, employing molecular clock models evaluated via nested sampling. The dates of sample collection were used as temporal calibration points on the leaf nodes of the tree. As part of the preliminary analysis, the nested sampling approach (NS) was used to compare various molecular clock models under three prior probability configurations, following the procedures described by Dyson et al. (37), Russel et al. (38), and Li et al. (16) (16, 37, 38). Combinations of strict and relaxed (log-normal) molecular clock models were evaluated alongside constant, exponential, and Bayesian Skyline Plot (BSP) coalescent demographic models, all assessed under three different prior probability configurations. For all model and state combinations, a chain length of 1,000,000 steps was used, with sampling every 5,000 steps and a burn-in of the first 100,000 steps. The optimal model, determined by the marginal likelihood value, was a relaxed log-normal molecular clock under a constant coalescent population scheme. The final analysis was performed with a chain length of 10,000,000 steps, with sampling every 5,000 steps and a burn-in of the first 1,000,000 steps. The resulting phylogeny was visualized using FigTree v1.4.4 (http://tree.bio.ed.ac.uk/software/figtree/).

## RESULTS

### Two independently established clonal lineages of STVM circulated simultaneously in Colombia

Among the 200 genomes analyzed, eight sequence types (STs) were identified. ST19 was the most prevalent, detected in 61.5% (*n* = 123) of sequences, followed by ST34 in 33.5% (*n* = 67). ST2379 and ST7478 were each identified in 1% (*n* = 2) of sequences, while ST7832, ST4920, ST3438, and ST3631 were detected in 0.5% (*n* = 1) of isolates. The ST could not be determined in two (1%) sequences (Fig. 1).

Phylogenetic and Bayesian analyses identified five clusters, with two well-defined Colombian lineages forming Clusters 1 and 2 (Fig. 1; Table 1; Fig. S1), collectively accounting for 85% (83/98) of the Colombian genomes (both human and swine chain) and characterized by ST19. Cluster 1 (Colombian lineage 1) is composed exclusively of 60 Colombian genomes: 28 from humans and 32 from pig farms and abattoirs. Cluster 2 (Colombian lineage 2, *n* = 27 genomes) was divided into two subclusters: the first comprised five international genomes from Brazil (*n* = 3), Canada (*n* = 1; ST3631), and Portugal (*n* = 1), together with one Colombian genome; the second comprised 22 Colombian genomes—16 from humans and six from pig farms and abattoirs. Bayesian analysis estimated that Colombian lineage 1 emerged around 1999, followed by rapid expansion and establishment between 2004 and 2005. In contrast, Colombian lineage two shares a Most Recent Common Ancestor (MRCA) with international genomes dating from approximately 1964, with stabilization in the country estimated at around 2003 (Fig. S1). These results suggest that Colombian lineages 1 and 2 have an independent origin from the predominant international STVM lineages circulating in Europe and the United States, which are grouped in Clusters 3 to 5. Clusters 3, 4, and 5 comprised international lineages as well as a subset of Colombian genomes. Cluster 3 (*n* = 70 genomes) was predominantly ST34 (96%), encompassing 13 Colombian genomes (10 human and three from pig farms and abattoirs), the reference genome of the European ST34 lineage (UK-EUROPE-SO4698-09), and 53 genomes from 11 different countries. The remaining 4% comprised genomes of ST2379 (*n* = 2) from Canada and the USA and ST7832 (*n* = 1) from Canada. Cluster 4 (*n* = 8 genomes) included the reference genome of the Spanish ST19 lineage (SPAIN-LSP-389-97) and 7 Canadian genomes, all assigned to ST19. Cluster 5 (*n* = 27 genomes) included one Colombian human genome (COL_021), 13 genomes from the USA, including the USA ST19 reference genome (USA-CVM23701), 12 Canadian genomes, and one Danish genome, all but one assigned to ST19. Additionally, the COL_035 ST19 monophasic variant 5-negative isolate was not grouped in any cluster (Fig. 1; Table 1).

**TABLE 1:** Comparative distribution of genomic and epidemiological features among STVM clusters^*a*^

Category of analysis	Cluster 1 (Colombian lineage 1)	Cluster 2 (Colombian lineage 2)	Cluster 3 (European lineage ST34)	Cluster 4 (Spanish lineage ST19)	Cluster 5 (American lineage ST19)
Number of genomes	60 Colombian 28 human 32 swine	27 genomes 22 Colombian: 16 human, six swine; five international: 3 Brazil 1 Canada 1 Portugal	70 genomes (13 Colombian: 10 human, three swine; 57 international)	8 genomes (1 Spanish reference, 7 Canadian)	27 genomes (1 Colombian human, 13 American, 12 Canadian, 1 Danish)
Estimated origin (year, country, MRCA)	Emerged around 1999, rapid expansion and establishment between 2004 and 2005 in Colombia.	Older origin (~1964), stabilization in Colombia since 2003. MRCA shared with international genomes.	Estimated lineage emergence ~1980. Reference strain is UK-EUROPE-SO4698-09.	First described in 1997. Reference strain is SPAIN-LSP-389-97.	First strain isolated in the USA in 1992. Reference strain is USA-CVM23701.
Reservoirs (human, animal, environmental)	Mainly humans and swine	Humans and swine in Colombia	Humans and swine in Colombia. Also animal, environmental	Clinical human isolates (Canadian) and livestock	Various sources (clinical humans, food, animals). The Colombian genome is human.
*fljAB* / STM2762–2773 deletion	85% lost the entire region between STM2762 and the 3 genes of the *fljAB* operon. Variable patterns, including conservation of some genes.	89.2% retained hin and deleted up to STM2762. Some (COL_001, Canada_30) retain the complete *fljAB* operon (atypical variants).	91% showed an extensive deletion from STM2762 to STM2772, consistent with the ST34 lineage.	Fewer deletions. The reference strain lacks the entire region up to STM2772. Other genomes with variable losses or conservation of the complete region.	Similar to Cluster 2, 89% retain the last gene of the *hin* operon. The American ST19 lineage retains part of the *fljAB* operon.
pSTV (virulence plasmid presence)	Highly frequent, suggested by IncFIB(S) and IncFII(S) and presence of virulence genes, such as *spvBCD*, *pefABCD*, *rck,* and *spvR*.	Highly frequent, suggested by IncFIB(S) and IncFII(S) and presence of virulence genes.	Absent, consistent with reports for this lineage.	Present, suggested by IncFIB(S) and IncFII(S).	Present, includes IncFIB-S, IncFII-S.
Plasmid incompatibility groups	IncFII-S (98.3%), Col-pHAD28 (53.3%), IncI1-I-Alpha (46.6%). Also IncFII-pHN7A8 and IncN. Various simultaneous presence profiles.	IncFII-S (82.1%), IncFIB-S (78.6%), IncFIB-AP001918 (60.7%), IncQ1 (42.9%). Total of 12 incompatibility groups.	16 Inc groups; in Colombian genomes, IncQ1 (77%) and Col-pHAD28 (69%) predominate.	IncFIB(S) and IncFII(S) identified in 85% of genomes in clusters 1, 2, 4, and 5, suggesting their presence. Specific distribution not detailed.	For COL_021: IncFIB-S, IncFII-S. Not detailed for the rest of the cluster.
Predominant prophages/prophage load	High load. Gifsy-2 (100%), Fels-2 (100%), Gifsy-1 (96.7%), UAB_Phi20 (95%). Also sal3, SW9, SP_004.	Lower and more diverse load. Gifsy-2 (100%), SP_004 (87%) is most characteristic. UAB_Phi20 in 11%. Gifsy-1 and Fels-2 not identified.	Combination of classic and atypical. sal3 (100%), Gifsy-1 (69.2%). Fels-2 and SW9 in lower proportion.	Not detailed in main text. (Metadata suggests Gifsy_1, Gifsy_2, Fels_2, UAB_Phi20.)	For COL_021: Gifsy-1, Gifsy-2, SW9. Not detailed for the rest of the cluster.
Key AMR genes/ resistance profile	Highly multidrug-resistant. *qnrB19, floR*, *tetA* predominate. Also *bla_CTX-M-55_*, *bla_TEM-141_*, *bla_OXA-10_*, *bla_OXA-2_*, *fosA3*, *ARR-3*, *qnrS1*, *dfrA14*, *tetD*, *lnuF*, *lnuG*.	Mainly *gyrA* (S83F/Y), *floR, tetB*, *sul2*, *bla_TEM-1B_*, *aadA2*, *aph (6)-Id*, *aph(3'')-Ib*, *aph(3'')-IIa*. *qnrB19* to a lesser extent.	Present *aph (6)-Id*, *aph(3'')-Ib*, *bla_TEM-1B_*, *sul2*, *tetB*. Local acquisition of *qnrB19* in Colombian strains.	Generally multidrug-resistant. Not detailed for this specific cluster.	Generally susceptible to antimicrobials.
Heavy metal resistance (operons/systems)	*arsRDABC* operon absent. Complete *merACPRT* operon. *copA*, *cusS* genes and *cueOR* system conserved.	*arsRDABC* operon absent. *merACPRT* operon shows deletion of *merT*. *copA*, *cusS* genes and *cueOR* system conserved.	Presents arsenic, mercury, copper resistance operons and the *cueOR* system.	Presence of *copA*, *cusS, cueO/R* and mer genes	Presence of *cueO/R, cus* and some mer genes
SPIs (pathogenicity islands)	Highly conserved (>90%): C63PI, CS54_island, SPI-1 to SPI-5, SPI-9, and SPI-11. SPI-12 absent.	Highly conserved (>90%): C63PI, CS54_island, SPI-1 to SPI-5, SPI-9, and SPI-11. SPI-12 absent.	Highly conserved (>90%). SPI-12 is more prevalent in this cluster.	Highly conserved (>90%). SPI-12 not detailed. (Metadata indicates SPI-12 absent.)	Highly conserved (>90%). SPI-12 is more prevalent in this cluster.
Additional virulence genes	*spvBCD, pefABCD, rck, mig-5, sopE2* (99% of genomes). *sopE* not identified.	*spvBCD, pefABCD, rck, mig-5, sopE2* (present in 78.6%-82.1% of genomes).	*pefABCD, spvBCD, mig-5,* and *rck* absent in almost all (except COL_030), consistent with absence of pSTV. *sopE* not detected in Colombian genomes of this cluster.	*spvBCD, pefABCD, rck, mig-5, sopE2,* are conserved.	*spvBCD, pefABCD, rck, mig-5, sopE2,* are conserved.
Biological, clinical, or environmental importance	Significant local adaptation. High load of resistance genes and plasmids. The presence of *qnrB19* and *bla_CTX-M-55_* is a concern. Match with previously reported clone reinforces local origin and dissemination capacity.	Older origin suggests local adaptation prior to the emergence of Lineage 1. Lower plasmid and resistance gene load than Lineage 1.	Coexistence with endemic lineages in Colombia. Evidence of local horizontal transfer (acquisition of *qnrB19* in Colombia).	Generalized multidrug-resistant clone.	Suggests multiple introduction routes to Colombia, possibly via trade.

^*a*^ Summary of key genomic characteristics distinguishing the two endemic phylogenetic clusters of STVM Colombia lineages 1 and 2, and the international lineages. Differences include prophage repertoire, antimicrobial resistance (AMR) determinants, heavy metal resistance genes, and mobile genetic elements.

The five cluster and Colombian lineages were further supported by the expanded phylogeny constructed with 603 genomes from the EnteroBase data set (Fig. S2; Table S3). Complementing this, a comprehensive pairwise core SNP distance analysis was conducted separately for each endemic Colombian lineage, providing quantitative support for the hypothesis of two independently established clonal lineages circulating simultaneously in the country. Within Cluster 1 (Colombian Lineage 1, *n* = 56 isolates), intracluster pairwise distances revealed a highly cohesive clonal structure, with a median of 48 SNPs across all pairs and a minimum of 0 SNPs between two human clinical isolates (COL_031 and COL_032), demonstrating the recovery of an identical clone from independent clinical cases. At the human–pig interface within Cluster 1, 39 pairs exhibited distances of ≤25 SNPs (consistent with prolonged interspecies transmission (34), including two pairs at only eight SNPs (COL_040-305-21 and SRR8740488 – 305-21), a threshold indicative of zoonotic transmission events (33). Notably, the single pig isolate 305-21 was genomically related (≤25 SNPs) to 10 distinct human clinical isolates, and the human isolate SRR8740488 shared ≤25 SNPs with 17 different pig isolates from diverse farms and abattoirs - a pattern only consistent with a clonal lineage that has been established and widely circulating at the human–pig interface for an extended period. Within Cluster 2 (Colombian Lineage 2, *n* = 20 isolates), pairwise human–pig distances were uniformly in the range of 15–25 SNPs (19 pairs ≤ 25 SNPs), with the closest pairs involving SRR8740536 and Pig-50/Pig-S2 (15 SNPs each), reflecting a pattern of prolonged transmission consistent with the earlier estimated stabilization date (~2003) of this lineage. Collectively, the within-cluster SNP analysis confirms that both Colombian endemic lineages exhibit sustained and independent zoonotic cocirculation between human clinical and pig reservoir isolates, without host-specific phylogenetic separation, and provides the quantitative genomic basis for classifying them as two distinct clonal lineages that have independently colonized the Colombian human–livestock interface.

The endemic Colombian lineages (Clusters 1 and 2) and the Colombian genomes associated with the European ST34 lineage in Cluster 3 circulated across 22 of 34 departments (administrative regions) of the country, without exclusive association with any particular geographical region. The phylogenetic analysis of this study can be accessed at the following link: https://microreact.org/project/eP8QohjSDKrKu6iWNN1ysS-genomiccomparativeSTVM200.

The expanded phylogenomic analysis including 603 STVM genomes confirmed that the isolates analyzed in this study do not form a single compact monophyletic cluster but instead are distributed among five well-supported phylogenetic lineages associated with distinct sequence types (ST19 and ST34) (Fig. 1; Fig. S2). This pattern indicates substantial genomic diversity within the monophasic variant and is consistent with previous reports describing the polyphyletic population structure of STVM (7, 22, 39, 40). Although our data set was designed to compare Colombian STVM isolates with representative international STVM lineages rather than to reconstruct the evolutionary transition from biphasic S. Typhimurium, the observed phylogenetic structure and the distinct *fljAB* deletion patterns identified across clusters are compatible with the hypothesis that the monophasic phenotype has arisen through multiple evolutionary trajectories (40). Demonstrating independent serovar-conversion events, however, would require a broader phylogenetic framework including representative biphasic *S*. Typhimurium genomes.

### The patterns of chromosomal deletions in the *fljAB* operon and surrounding genes vary across Colombian lineages

A conserved feature of international STVM lineages is the deletion of the genomic region flanked by the STM2762 and STM2773 genes, located downstream and upstream of the *fljAB* operon, respectively. To determine the deletion patterns, the Colombian genomes were compared with the *S*. Typhimurium LT2, European ST34, Spanish ST19, and USA ST19 reference genomes. Distinct deletion patterns were identified across each of the five clusters defined in the preceding analysis.

**Fig 2:**
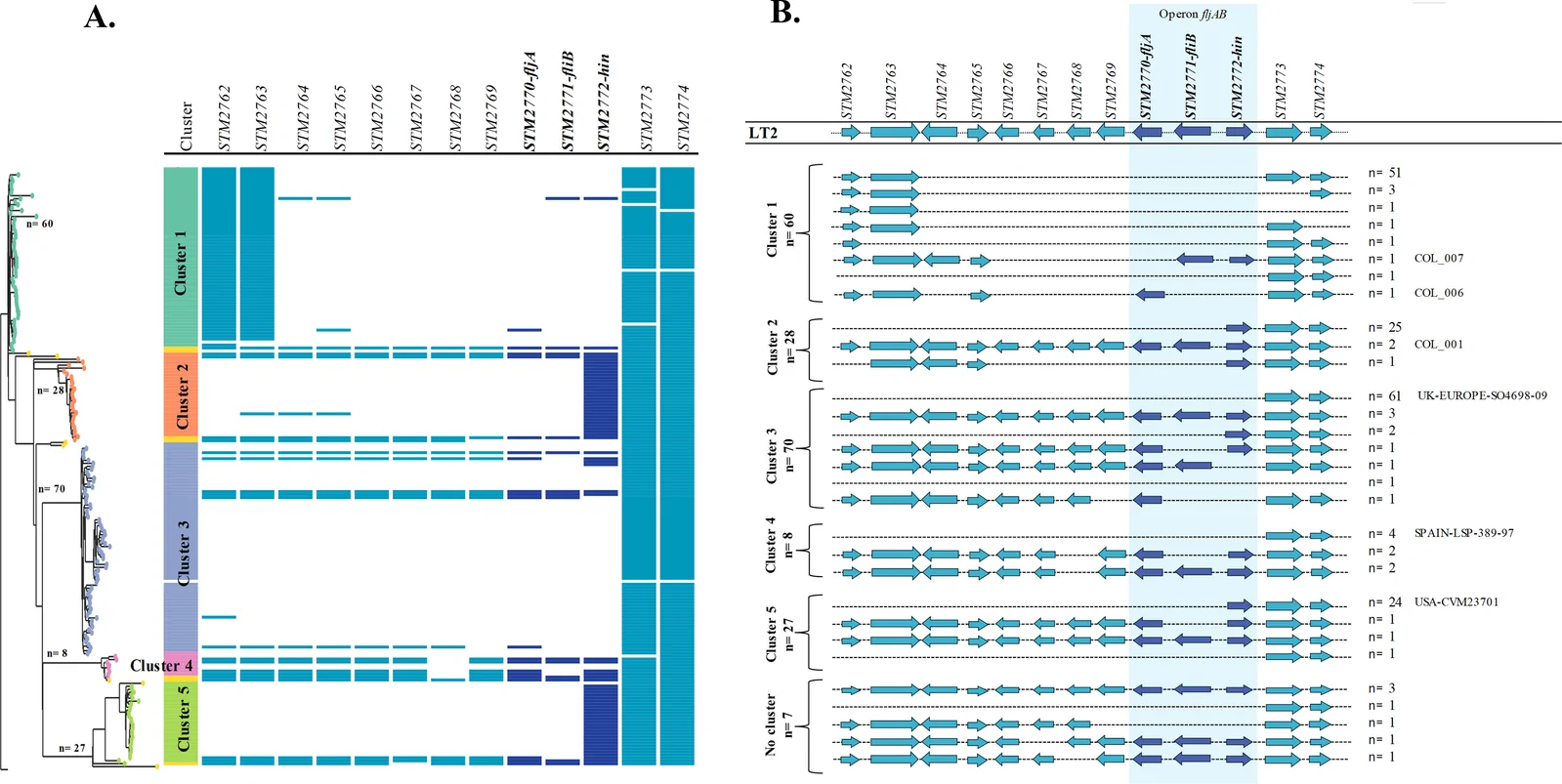
Deletion patterns of the *fljAB* operon and flanking genes. (**A**) Maximum-likelihood SNP-based phylogenetic tree of 200 STVM genomes, grouped into five main clusters: turquoise (Cluster 1), salmon (Cluster 2), purple (Cluster 3), pink (Cluster 4), and green (Cluster 5). Genomes not assigned to these clusters are shown in light yellow (“no cluster”). The central matrix depicts the presence (dark blue, full boxes) or absence (white, empty boxes) of the genes spanning STM2762–STM2774. The three core genes of the *fljAB* operon (STM2770–*fljA*, STM2771–*fljB*, and STM2772–*hin*) are shown in dark blue when present in full length; surrounding genes (STM2762–STM2769 and STM2773–STM2774) are shown in light blue. White boxes indicate gene absence. (**B**) Genomic context of the *fljAB* operon in the reference strain *S*. Typhimurium LT2, and representative deletion patterns for each phylogenetic cluster. Genes are represented as arrows indicating transcriptional orientation, with the *fljAB* operon highlighted by a shaded blue background. brackets indicate the cluster and the number of genomes (**N**) displaying each pattern. Reference strains are labeled (e.g., UK-EUROPE-S04698-09, SPAIN-LSP-389-97, USA-CVM23701). Genomes not assigned to any cluster are grouped under “no cluster.”

In the Colombian lineages (Clusters 1 and 2), the chromosomal deletion patterns differed in the region between STM2762 and *iroB* (STM2773) (Fig. 2). In Colombian lineage 1 (Cluster 1), 85% (*n* = 51/60) of genomes exhibited loss of the entire region between STM2762 and the three genes of the *fljAB* operon (STM2770-*fljA*, STM2771-*fljB*, and STM2772-*hin*). Four genomes (6.7%) showed variable deletion patterns that conserved some operon genes, and one genome (1.6%) conserved all three operon genes but lacked the downstream region from STM2762 to STM2769, constituting an atypical variant. In contrast, Colombian lineage 2 (Cluster 2) was characterized by conservation of the *hin* gene of the *fljAB* operon alongside deletion of the entire upstream region up to STM2762 in 89.2% (*n* = 25/28) of genomes; two genomes retaining the complete *fljAB* operon (Canada_30 and COL_001) were classified as atypical variants. In particular, COL_001 preserves the second flagellar phase at the genetic level, although it is not expressed serotypically (15). One genome (COL_043) conserved only three genes within the entire region (STM2763 to STM2765).

A more extensive deletion pattern was observed in Cluster 3 (*n* = 70), composed exclusively of ST34 genomes, including 13 genomes from human clinical isolates and Colombian pig farms and abattoirs. In 91% (*n* = 64/70) of these genomes, the deletion spanned from STM2762 to STM2772, consistent with reports for monophasic variants of ST34 and the reference strain UK-EUROPE-SO4698-09.

Genomes in Cluster 4 (*n* = 8) exhibited fewer deletions than those in the other clusters. This group includes the reference strain SPAIN-LSP-389-97 of the Spanish ST19 lineage, which lacks the entire analyzed region up to STM2772, and three genomes that lost the entire region up to the *fljAB* operon. The remaining four genomes conserved the entire region from STM2762, including the *fljAB* operon, with only sporadic gene losses (Fig. 2).

Finally, Cluster 5 showed considerable similarity to Cluster 2, with 89% of genomes (*n* = 24/27) retaining the *hin* gene. This result was expected, as this cluster includes the USA ST19 reference strain USA-CVM23701 and comprises predominantly North American genomes (41) Notably, the COL_021 genome represents the only Colombian human clinical isolate within this cluster (Fig. 2).

### The Colombian lineages of STVM harbor distinct accessory genome contents relative to international lineages

**Fig 3:**
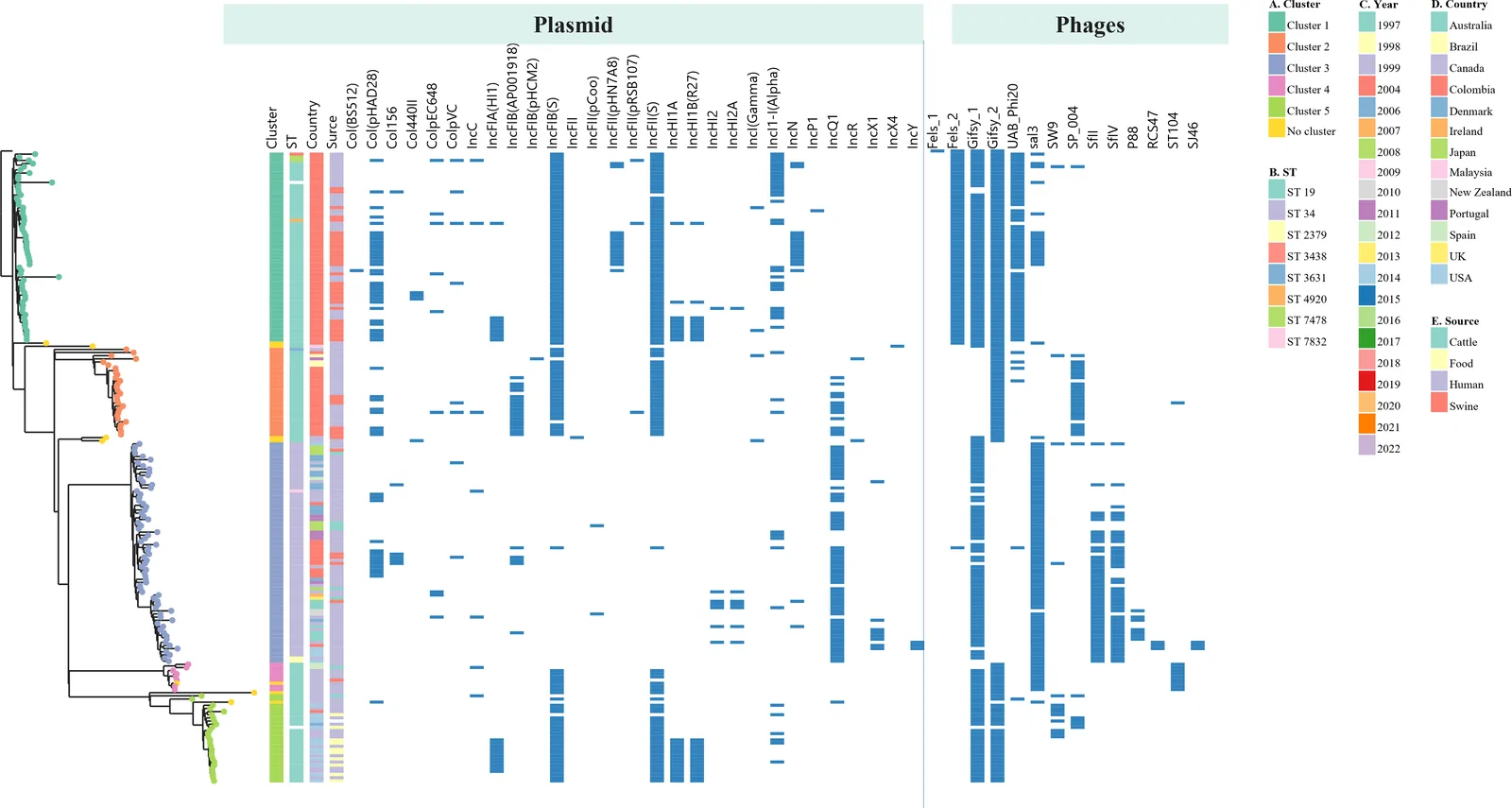
Acquisition of plasmid and prophage genetic elements. Maximum-likelihood SNP-based phylogenetic tree of Colombian and international STVM genomes, annotated with the presence/absence profiles of plasmid (left section) and prophage (right section) genetic elements. Columns on the right are grouped by element type (“plasmid” and “phages”), with each column representing a specific acquired genetic element (name indicated at the top). Dark blue cells indicate the presence of the element, while white cells indicate its absence. The columns on the far right display the color code for *Cluster*, *ST*, *Country*, and *Source*, as detailed in the adjacent legend.

Twenty-nine incompatibility groups (Inc) of virulence or resistance plasmids were identified in the 200 genomes analyzed. In the Colombian genomes, between 1 and 12 Inc groups were identified per genome. The IncFIB(S) and IncFII(S) groups were identified in 85% (*n* = 84/98) of the genomes in Clusters 1, 2, 4, and 5. These groups suggest the presence of the Typhimurium virulence plasmid (pSTV), and their detection coincides with the presence of the virulence genes *spvBCD*, *pefABCD*, and *rck*, as described below. Furthermore, the detection of the *spvR* regulatory gene, also carried by pSTV, reinforces the identification of this plasmid in Colombian lineages. The second most prevalent group was ColpHAD28, present in 50% (*n* = 49/98) of genomes in Clusters 1, 2, and 3. The third most common group was IncI1-I alpha, identified in 33% (*n* = 32/98) of genomes (Fig. 3).

The distribution of Inc groups differed across the Colombian lineages. Colombian lineage 1 (Cluster 1) was notable for the Inc groups IncFII-S (98.3%, *n* = 59/60), Col-pHAD28 (53.3%, *n* = 32/60), IncI1-I-Alpha (46.6%, *n* = 28/60), IncFII-pHN7A8 (23.3%, *n* = 14/60), and IncN (*n* = 14/60), as well as two simultaneous plasmid presence profiles: the first - IncFIB-S, IncFII-S, IncI1-I-Alpha - in 23% (*n* = 14/60) of genomes, and the second - Col-pHAD28, IncFIB-S, IncFII-pHN7A8, IncFII-S, IncN - in 18% (*n* = 11/60) of genomes (Fig. 3).

Colombian lineage 2 (Cluster 2) exhibited 12 Inc groups, with IncFII-S (82.1%, *n* = 23/28), IncFIB-S (78.6%, *n* = 22/28), IncFIB-AP001918 (60.7%, *n* = 17/28), and IncQ1 (42.9%, *n* = 12/28) being the most prevalent. The MDR isolate COL_009 carried 10 of the identified Inc groups, while four pig genomes and one human genome each carried five Inc groups (see Table S1—Metadata). Cluster 3, containing 13 Colombian genomes grouped with the European ST34 lineage, harbored 16 Inc groups, of which 8 were present in the Colombian genomes; the most prevalent were IncQ1 (77%, *n* = 10/13) and Col-pHAD28 (69%, *n* = 9/13). Notably, the pSTV-associated groups were not identified in this cluster, consistent with what has been reported for this lineage (7). The sole Colombian genome in Cluster 5 (COL_021) carried only the pSTV-associated groups described above (IncFIB-S and IncFII-S) (Fig. 3).

Fourteen distinct prophage regions were identified across the data set (Fig. 3). Most genomes harbored between 5 and 12 prophages, with a high prevalence of Gifsy-1 (81%, *n* = 162), Fels-2 (80.5%, *n* = 161), UAB_Phi20 (69%, *n* = 138), and Gifsy-2 (66%, *n* = 132). Less frequent prophages were also detected, including SP_004 in 23% (*n* = 46), SW9 in 21% (*n* = 42), sal3 in 19% (*n* = 38), ST104 and RCS47 in 5% (*n* = 10), SfII in 3.5% (*n* = 7), P88 and SJ46 in 1.5% (*n* = 3), and Fels_1 in one genome.

Among the 98 Colombian genomes, Colombian lineage 1 (Cluster 1) carried Gifsy-2 and Fels-2 in 100% of genomes, Gifsy-1 in 96.7% (*n* = 58/60), and UAB_Phi20 in 95% (*n* = 57/60). Furthermore, sal3 was detected in 26.7% (*n* = 16/60) of genomes, and SW9 and SP_004 each in one genome. Colombian lineage 2 (Cluster 2), by contrast, exhibited a lower burden of classical prophages and a more diverse distribution. Neither Gifsy-1 nor Fels-2 was identified in this cluster. However, Gifsy-2 was present in 100% of genomes, while UAB_Phi20 was detected in only three isolates (11%). The prophage SP_004 was found in 87% (*n* = 20/23) of genomes, making it the most characteristic element of this cluster.

The 13 Colombian genomes associated with the European ST34 lineage harbored a combination of classical and atypical prophage elements: Gifsy-1 was detected in 69.2% (*n* = 9/13), sal3 in 100% (*n* = 13/13), and Fels-2 and SW9 each in 7.7% (*n* = 1/13). The single Colombian genome in Cluster 5 carried Gifsy-1, Gifsy-2, and SW9. The mTmV prophage was absent in all Colombian isolates.

**Fig 4:**
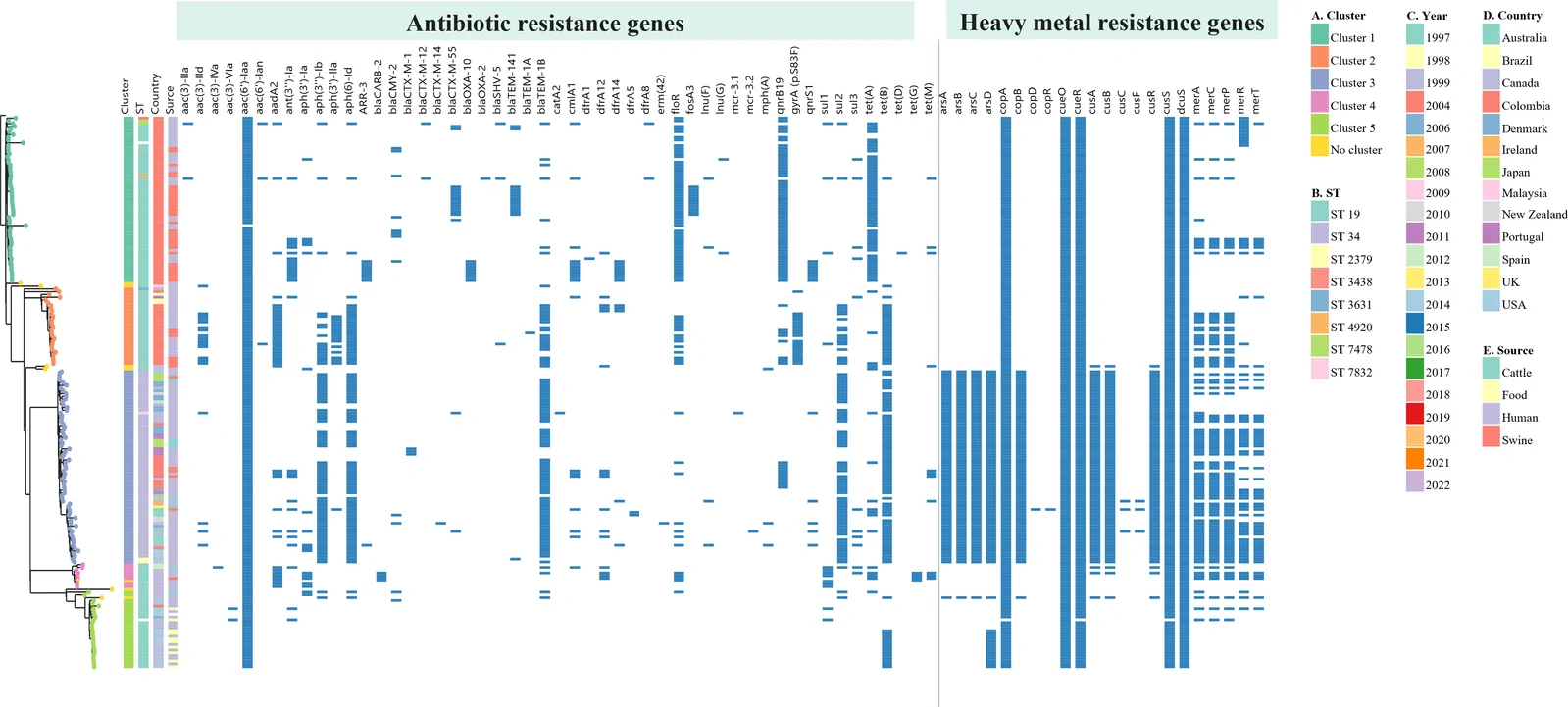
Acquisition of genetic elements conferring resistance to antimicrobials and heavy metals. Maximum-likelihood SNP-based phylogenetic tree of Colombian and international STVM genomes, annotated with the presence/absence profiles of genes associated with antibiotic resistance (left section) and heavy metal resistance (right section). Dark blue cells indicate the presence of the gene, while white cells indicate its absence. The columns on the far right display the color code for *Cluster*, *ST*, *Country*, and *Source*, as detailed in the adjacent legend.

A total of 49 antimicrobial resistance genes belonging to nine antibiotic families were identified in the Colombian genomes, as shown in Fig. 4. The *qnrB19* gene, conferring resistance to fluoroquinolones, was the most prevalent at 74.5% (*n* = 73/98), followed by the *floR* gene (chloramphenicol resistance) at 72.4% (*n* = 71/98), *tetA* (tetracycline resistance) at 58.2% (*n* = 57/98), *tetB* at 39.7% (*n* = 36/98), and *bla*TEM-1B (resistance to penicillins, cephalosporins, and aztreonam) at 36.7% (*n* = 36/98). Additional resistance genes were detected in fewer than 50% of genomes; the most frequent were *aph (6)-Id* (35.7%; 35/98), *aadA2* (28.6%; 28/98), *aph(3″)-Ib* and *sul2* (26.5%; 26/98), *gyrA* with the S83F mutation (19.4%; 19/98), and *dfrA14* (11.2%; 11/98). Several additional β-lactam resistance genes were identified at lower frequencies (range 1–14.3%), including *blaOXA-2*, *blaTEM-1A*, *blaCTX-M-12*, *blaSHV-5*, *blaOXA-10*, *blaCMY-2*, *blaTEM-141*, and *blaCTX-M-55*. Notably, the *lnuF* and *lnuG* genes, which confer resistance to lincosamides, were detected in 2% of isolates.

Phylogenetic grouping revealed differences in resistance gene content among Colombian lineages 1 and 2 and the European ST34 lineage. In Colombian lineage 1, the genes *qnrB19*, *floR*, and *tetA* were primarily found, in addition to *bla*CTX-M-55, *bla*TEM-141, *bla*OXA-10, *bla*OXA-2, *fosA3*, ARR-3, *qnrS1*, *dfrA14*, *tetD*, *lnuF*, and *lnuG*. In contrast, Colombian lineage 2 mainly carried *gyrA*, *floR*, *tetB*, *sul2*, *bla*TEM-1B, *aadA2*, *aph (6)-Id*, *aph(3″)-Ib*, *aph(3″)-IIa*, and other aminoglycoside resistance genes, with *qnrB19* present to a lesser extent. Colombian genomes grouped with the European ST34 lineage carried *aph (6)-Id*, *aph(3'')-Ib*, *bla*TEM-1B, *sul2*, and *tetB*, in common with other genomes of Cluster 3. Notably, *qnrB19* carriage in Colombian genomes was not observed in genomes from other geographic origins (Fig. 2; Table S1 - Metadata).

Regarding heavy metal resistance genes, their acquisition through horizontal gene transfer is a well-established characteristic of STVM isolates and an important feature of this variant (41). In this study, resistance operons for arsenic (*arsRDABC*), copper (*copABDR*), and mercury (*merACPRT*), as well as copper homeostasis systems (*cueOR* and *CusABCFRS*), were identified. These results revealed differences in gene content between the Colombian lineages and the European ST34 lineage (Fig. 2). In Colombian lineages 1 and 2, the arsenic resistance operon was absent; the mercury resistance operon was complete in lineage 1, whereas lineage two exhibited deletion of *merT*. With respect to copper resistance systems, the *copA* and *cusS* genes, as well as the *cueOR* system, were conserved in both lineages. The isolates of the European ST34 lineage (Cluster 3), in contrast, harbored resistance operons for arsenic, mercury, and copper along with the *cueOR* system, consistent with previously reported characteristics of this lineage.

### Virulence genes in the STVM

Ten main pathogenicity islands (SPIs) were identified in the 200 genomes analyzed: C63PI, CS54_island, SPI-1 to SPI-5, SPI-9, SPI-11, and SPI-12. The core virulence islands—C63PI, SPI-1 to SPI-5, SPI-9, and SPI-11 - were highly conserved, with a prevalence exceeding 90% in most clusters, reflecting their essential role in STVM pathogenesis. CS54_island was also prevalent (91.5%), while SPI-12 showed a limited distribution (22%), with a greater presence in Clusters 3 and 5.

Among the 98 Colombian genomes analyzed, the main repertoire of pathogenicity islands was highly conserved. The islands C63PI, CS54_island, SPI-1 to SPI-5, SPI-9, and SPI-11 were present in more than 90% of the genomes, reflecting a stable and functional virulence repertoire. In contrast, SPI-12 was absent in all Colombian isolates.

The virulence genes *rck* and the *spvBCD* and *pefABCD* operons, encoded on the pSTV plasmid, were detected in 85 of the 98 Colombian genomes, with predominant representation in lineages 1 (*n* = 60) and 2 (*n* = 28). In Colombian lineage 1 (Cluster 1), 99% of genomes carried nearly all genes of these operons, reflecting high plasmid conservation. Similarly, the *mig-5* gene was detected in 57 of the 60 genomes. The *sopE2* gene, encoding an effector of the type III secretion system, was present in all 60 genomes of this cluster, while *sopE* was not identified in any genome in this study. Colombian lineage 2 (Cluster 2) displayed a similar pattern, with pSTV genes detected in 22 to 23 genomes; *sopE2* was found in 23 of 28 genomes, and *mig-5* in 20 of 28.

In Cluster 3, among the 13 Colombian genomes, pSTV-associated virulence genes were conserved only in the COL_030 genome. The *pefABCD*, *spvBCD*, *mig-5*, and *rck* genes were absent in nearly all genomes of this cluster, consistent with the absence of pSTV in the European ST34 lineage, as previously reported (7). Notably, the *sopE* gene, associated with international epidemic lineages such as ST34, was not detected in any Colombian genome of Cluster 3. Cluster 5 (*n* = 1; COL_021) also conserved all genes of interest, including the *spv* and *pef* operons and the *rck*, *mig-5*, and *sopE2* genes.

## DISCUSSION

Overall, our analyses identified five phylogenetic clusters, with Colombian isolates predominantly grouped into two lineages that are distinct from international ones. The distinct profiles of antibiotic resistance genes, prophages, and plasmids in each Colombian lineage, together with specific deletions in the *fljAB* operon, point to divergent evolutionary trajectories between the two lineages. Colombian Lineage 1 (Cluster 1) appears to have emerged recently (MRCA ~1999) and expanded throughout the population between 2004 and 2005. It is characterized by the retention of all four classical prophages (Gifsy-2, Gifsy-1, Fels-2, and UAB_Phi20), while 85% of its genomes exhibit loss of the entire region between STM2762 and the three *fljAB* operon genes. According to Yang et al. (42), Colombian Lineage 1 (Cluster 1) evolved through micro-deletions rather than block loss. This distinction is particularly noteworthy because prophage-encoded genes ineclude virulence factors and fitness determinants; the conservation of a larger prophage repertoire in Lineage one may therefore confer adaptive advantages in both clinical and livestock environments. Indeed, a notable collection of antimicrobial resistance genes (*qnrB19*, *floR*, and *tetA*) and heavy metals resistance operons (*mer*) are harbored on a diverse array of plasmids. The presence of the quinolone resistance gene *qnrB19* on extended-spectrum plasmids and of β-lactamases such as *bla*CTX-M-55 is of particular concern, as these mobile elements facilitate the spread of resistance to critically important drugs (43). The possible association of this lineage with clinical settings is further suggested by the presence of the rare genes *lnuF* and *lnuG*, which have been identified in strains of *Salmonella* and other *Enterobacteriaceae* conferring resistance to lincosamides (44). Its coincidence with Cluster seven described by Li et al. (16) reinforces its local origin and capacity for international dissemination.

In contrast, Colombian Lineage 2 (Cluster 2) has an earlier evolutionary origin (MRCA ~1964) and appears to have stabilized in Colombia around 2003. This cluster displays a distinct profile of antibiotic resistance genes, plasmids, and prophage content, with three of the four classical prophages (Gifsy-1, Fels-2, and UAB_Phi20) absent — mirroring the extensive genomic streamlining observed in internationally dominant STVM lineages such as the European ST34 — and with approximately 90% of genomes exhibiting block-level loss of 10 genes from STM2762 to *fljB* while conserving the *hin* gene. Fluoroquinolone resistance in Lineage two is mediated by a chromosomal mutation in *gyrA* (S83F/Y), and the overall burden of plasmids and resistance genes is lower. According to Yang et al. (42), these findings are consistent with a block-loss behavior rather than microdeletions. These characteristics are also consistent with those described by Li et al. (16), who identified this group as Cluster 5 (ST19-MDR). The earlier introduction of this lineage suggests that it had adapted locally before the emergence of Lineage 1.

A notable observation is that the Colombian lineages lack the mTmV prophage while retaining the Gifsy-2 region. This pattern may be interpreted as a trade-off between early intestinal invasion potential and intracellular resilience (7, 45). The epidemic European monophasic clone expanded clonally after multiple independent acquisitions of the mTmV prophage carrying the *sopE* gene, and this event has been associated with increased epidemiological success and transmission (7, 40). *SopE* is a SPI-1 type III secretion effector that enhances trigger-mediated invasion of intestinal epithelial cells and promotes disruption of the brush border and barrier function, thereby facilitating rapid intestinal colonization and diarrhea (45, 46). Consequently, loss of mTmV typically entails loss of *sopE*, which may attenuate the competitive advantage of these lineages during the initial epithelial invasion phase in the gut. In contrast, conservation of Gifsy-2 preserves the prophage-encoded *sodC1* gene, which encodes a periplasmic Cu,Zn-superoxide dismutase that contributes to detoxification of reactive oxygen species and enhances survival of *Salmonella* within phagocytic cells and in murine infection models (47, 48). Thus, Colombian STVM lineages that lack mTmV-*sopE* but retain Gifsy-2-*sodC1* may display reduced epidemic “explosiveness” compared with the dominant European ST34 clone, yet remain clinically relevant threats due to a conserved virulence core and robust intracellular survival in the face of the host oxidative burst.

The distinctiveness of the two Colombian endemic lineages is further supported by their international genomic contexts revealed in the expanded EnteroBase analysis (Fig. S2). Whereas Cluster 1 (Colombian Lineage 1) has no close international counterparts in the 603-genome expanded data set and is recovered as a fully Colombian-exclusive monophyletic clade, Cluster 2 (Colombian Lineage 2) exhibits notable genomic proximity to Brazilian isolates (SRR7066544, SRR7066581, SRR7064866; 6–9 SNPs), indicating a shared recent common ancestor with South American strains rather than with the European ST34 or Spanish ST19 lineages. This South American connection suggests that the establishment of Lineage two in Colombia may represent part of a broader regional dissemination event within Latin America, predating the emergence of Lineage 1. Taken together with the within-cluster SNP distances, these findings provide three convergent lines of genomic evidence—phylogenetic topology, Bayesian divergence dating, and pairwise SNP distances—that collectively establish the co-circulation of two independently established endemic STVM clones in Colombia. Notably, although these lineages stabilized between 2003 and 2005, they were not identified by serotyping or reported until 2015 by the Acute Diarrheal Disease (ADD) laboratory surveillance system of the National Institute of Health. This temporal gap suggests that the lineages circulated without formal characterization for more than a decade, highlighting the importance of strengthening genomic surveillance as a complement to traditional methods.

The high prevalence of resistance to tetracycline, chloramphenicol, fluoroquinolones, and ampicillin in both Colombian lineages likely reflects the selective pressure derived from the historical and current use of these antibiotics in national animal production (49). Furthermore, the widespread presence of genes for resistance to heavy metals, including those conferring resistance to mercury, arsenic, copper, and zinc, suggests active co-selection processes in contaminated environments, particularly in agricultural settings where these metals are commonly used. Differential resistance profiles between lineages indicate possible local adaptations and reinforce the hypothesis that heavy metals may contribute to the persistence of multidrug-resistant strains (MDRs) in the environment (43, 50).

An additional key finding of this study is the close phylogenetic relationship between isolates of human and porcine origin, indicative of active and sustained interspecies transmission. The two endemic Colombian lineages identified (Clusters 1 and 2) accounted for 85% of the genomes analyzed, with no differentiated phylogenetic structure by host species, underscoring the swine production chain as a fundamental axis in the epidemiology of STVM in Colombia.

In addition to the endemic Colombian lineages, internationally relevant lineages were detected. Notably, the European ST34 lineage (Cluster 3) circulates in Colombia in both humans and the swine production chain; however, its acquisition of the *qnrB19* gene, absent in European counterparts, suggests local adaptation through horizontal gene transfer and the potential emergence of distinct resistance profiles. Similarly, the American ST19 lineage (Cluster 5) was detected, suggesting multiple routes of introduction, possibly related to trade in meat products (51, 52).

The genomic characterization of the monophasic variant of *S*. Typhimurium (4,[5],12:i:-) (STVM) underscores a consistent global pattern: independent endemic lineages emerge through local adaptation alongside internationally circulating clones, a phenomenon documented in Australia, the United States, and multiple European countries (53). These regional adaptations contrast with the initial global dissemination driven by the European ST34 epidemic lineage (Cluster 3), which achieved success through early acquisition of mobile genetic elements conferring resistance to heavy metals (e.g., SGI-4 for copper/arsenic and mercury) and the multidrug resistance ASSuT profile (40, 52, 54). The collective genomic data confirms that the global establishment and expansion of STVM have been driven by multiple independent appearance events worldwide, fostering adaptation to specific regional ecological niches under varying selective pressures related to antimicrobial resistance and heavy metal usage.

The importance of applying the “One Health” approach to better understand the evolutionary dynamics of monophasic variant of *S*. Typhimurium (4,[5],12:i:-) is evident. The industrialization and centralization of pig production in Latin America, paralleling processes that have occurred in Europe, have been identified as factors driving its expansion. Pigs serve as important reservoirs, facilitating cross-host transmission including humans. The remarkable genomic plasticity of the STVM, reflected in the emergence of endemic lineages and their cocirculation with international ones, together with a high burden of resistance genes and mobile elements, underscores its adaptive capacity and potential for interspecies dissemination. Genomic epidemiology allows for the precise detection of these dynamics, revealing patterns of transmission and evolution that traditional surveillance systems cannot capture. It is therefore essential to implement integrated, sustained, and active genomic surveillance systems capable of anticipating emerging threats, tracing transmission routes, and informing effective control strategies under the One Health approach.

### Study limitations

The present analysis is constrained by the sample size and the narrow temporal window of the 98 Colombian genomes analyzed, which limited our ability to assess multi-year persistence and clonal replacement patterns of these Colombian STVM lineages. Future longitudinal studies incorporating more recent genomic data are warranted to refine our understanding of the spatiotemporal dynamics and adaptive landscape of STVM within the Colombian context. A second limitation is the targeted approach to international genome selection, which—while enabling direct comparison with well-characterized international lineages (European ST34, Spanish ST19, and USA ST19)—may not capture the full global diversity of STVM. Consequently, conclusions regarding the independent origin of Colombian lineages and divergence time estimates should be interpreted within this constraint. Additionally, deletion patterns in the *fljAB* region were determined through comparative analysis of assembled genomes rather than read mapping, which would provide additional validation. Future studies incorporating broader genome sampling and formal tests for positive selection would strengthen phylogeographic inferences and adaptive evolution hypotheses.

### Conclusions

This study identified two distinct endemic lineages (Cluster one and Cluster 2) of the monophasic variant of *S*. Typhimurium (4,[5],12:i:-) in Colombia, recovered from both pigs and humans. Phylogenomic analysis suggests that these lineages emerged in the country around 1999. These endemic lineages coexist with STVM international lineages included in this study. The genomic analysis demonstrated a close phylogenetic relationship between isolates of human and porcine origin within the endemic Colombian lineages (Clusters 1 and 2), revealing active and sustained interspecies transmission. The absence of clear phylogenetic separation by host species indicates active zoonotic cocirculation and closely integrated interspecies transmission dynamics, highlighting the swine production chain as a fundamental axis in the epidemiology of STVM in Colombia (55). The findings underscore the critical need for integrated surveillance strategies applied under the “One Health” framework, which mandates the combination of human, veterinary, and environmental monitoring. Strengthening genomic surveillance in parallel with traditional methods is essential to effectively anticipate the emergence of high-risk clones, trace transmission routes, mitigate their dissemination, and inform public health interventions.

## Data Availability

Whole-genome sequencing data for Colombian clinical isolates are deposited in the NCBI SRA under BioProject PRJNA527650 (*Salmonella* Typhimurium and 4,[5],12:i:- isolates from Colombia, 1999-2017), obtained through the collaboration of the Instituto Nacional de Salud with the *Salmonella* 10,000 Genomes Project at the University of Liverpool. Whole-genome sequencing data for the remaining Colombian clinical isolates will be deposited under BioProject PRJNA1516634. Genomic sequences from Colombian pig isolates were obtained from previously published studies (14) (I. Barrientos, A. K. Carrascal, A. Pulido-Villamarín, I. Chamorro, L. C. Veloza, M. Wiesner, F. Rojas, M. Pérez, J. E. Olsen, Y. Hounmanou, A. Dalsgaard, L. Alban, unpublished data); sequences are deposited in ENA under BioProject PRJEB80816 and will be publicly available upon publication. International reference genomes were retrieved from EMBL-EBI and EnteroBase as described in Materials and Methods. Phylogenetic trees are publicly accessible via Microreact at https://microreact.org/project/eP8QohjSDKrKu6iWNN1ysS-genomiccomparativestvm200 and https://microreact.org/project/5zJKTrm94gQTkDGiMvGoQp-newenterobasetree.

## SUPPLEMENTAL MATERIAL

Fig. S1 spectrum.03870-25-s0001.pdf Time phylogeny analysis of Colombian vs international STVM isolates.

Fig. S2 spectrum.03870-25-s0002.pdf Global phylogenetic context of Colombian *Salmonella* Typhimurium monophasic variant (STVM) genomes retrieved from Enterobase.

Supplemental legends spectrum.03870-25-s0003.docx Legends for Fig. S1 and S2.

Table S1 spectrum.03870-25-s0004.xlsx Genomic and epidemiological metadata of 98 Colombian and 102 international *Salmonella* Typhimurium monophasic variant (STVM) isolates included in the phylogenomic analysis.

Table S2 spectrum.03870-25-s0005.xlsx Genomic metadata and phylogenetic cluster assignment of 603 *Salmonella* Typhimurium monophasic variant genomes from the expanded EnteroBase-based global phylogenomic analysis.

Table S3 spectrum.03870-25-s0006.xlsx Pairwise core SNP distance matrix of STVM genomes from the expanded EnteroBase dataset (603 genomes), including intracluster distances for Colombian endemic lineages (Cluster 1, *n* = 56; Cluster 2, *n* = 20) and intercluster distances between Cluster 2 and the closest international genomes.

ASM does not own the copyrights to Supplemental Material that may be linked to, or accessed through, an article. The authors have granted ASM a non-exclusive, world-wide license to publish the Supplemental Material files. Please contact the corresponding author directly for reuse.
