# Supplementary material for: Comparative genomics of the monophasic variant of Salmonella Typhimurium: analysis of Colombian genomes and their relationship with international lineages
Source: Microbiol Spectr. 2026 Sep 17;14(10):e03870-25. doi: 10.1128/spectrum.03870-25 (PMC13689331; doi:10.1128/spectrum.03870-25)
Supplement: Supplementary file 1 [file spectrum.03870-25-s0001.pdf]

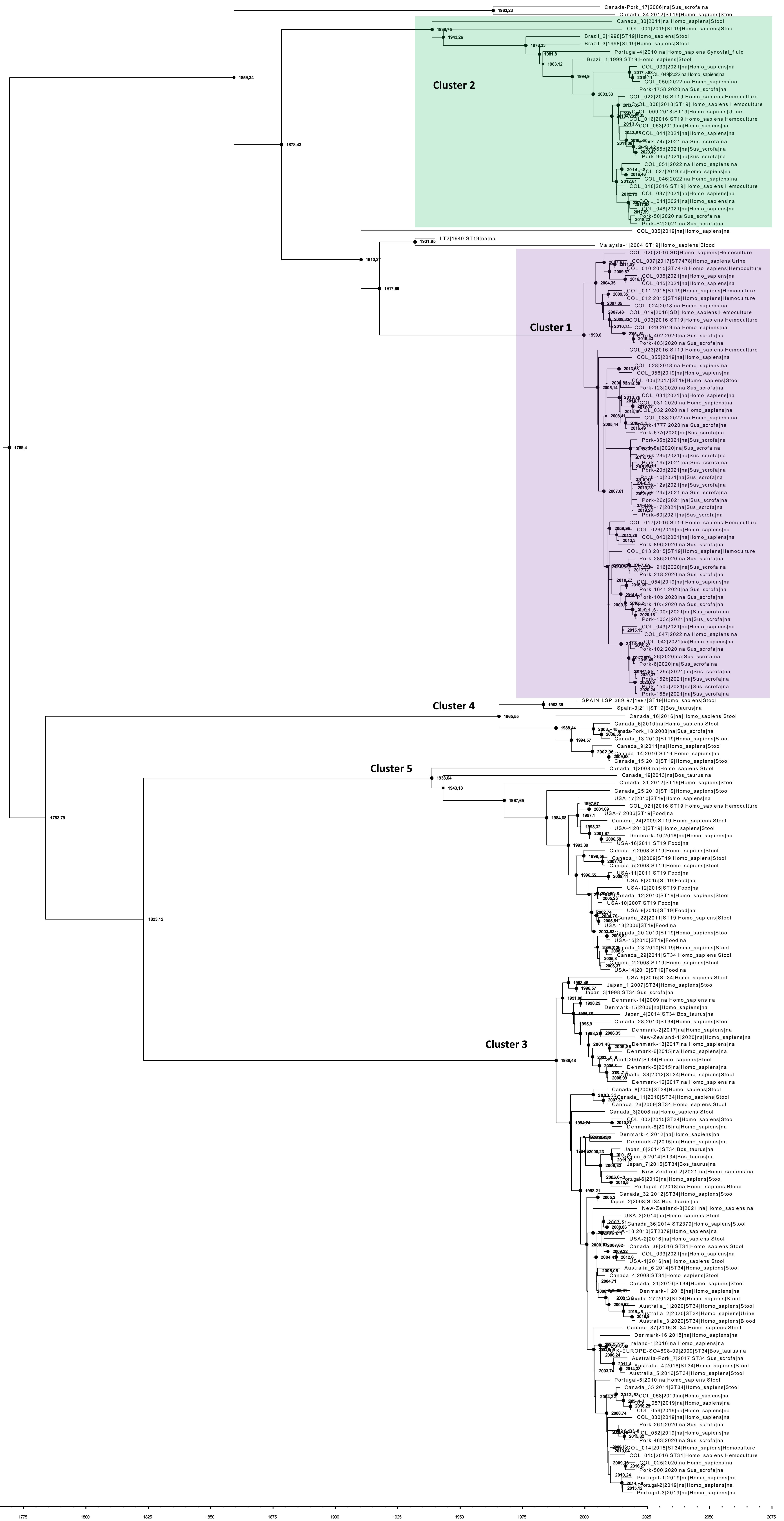
Canada-Pork_17|2006|na|Sus_scrofa|na
Canada_34|2012|ST19|Homo_sapiens|Stool
Canada_30|2011|na|Homo_sapiens|Stool
COL_001|2015|ST19|Homo_sapiens|Stool
Brazil_2|1998|ST19|Homo_sapiens|Stool
Brazil_3|1998|ST19|Homo_sapiens|Stool
Portugal-4|2010|na|Homo_sapiens|Synovial_fluid
Brazil_1|1999|ST19|Homo_sapiens|Stool
Cluster 2
COL_039|2021|na|Homo_sapiens|na
COL_049|2022|na|Homo_sapiens|na
COL_050|2022|na|Homo_sapiens|na
Pork-1758|2020|na|Sus_scrofa|na
COL_022|2016|ST19|Homo_sapiens|Hemoculture
COL_008|2018|ST19|Homo_sapiens|Hemoculture
COL_009|2018|ST19|Homo_sapiens|Urine
COL_016|2016|ST19|Homo_sapiens|Hemoculture
COL_053|2019|na|Homo_sapiens|na
COL_044|2021|na|Homo_sapiens|na
Pork-74c|2021|na|Sus_scrofa|na
Pork-65d|2021|na|Sus_scrofa|na
Pork-96a|2021|na|Sus_scrofa|na
COL_051|2022|na|Homo_sapiens|na
COL_027|2019|na|Homo_sapiens|na
COL_046|2022|na|Homo_sapiens|na
COL_018|2016|ST19|Homo_sapiens|Hemoculture
COL_037|2021|na|Homo_sapiens|na
COL_041|2021|na|Homo_sapiens|na
COL_048|2021|na|Homo_sapiens|na
Pork-50|2020|na|Sus_scrofa|na
Pork-S2|2021|na|Sus_scrofa|na
COL_035|2019|na|Homo_sapiens|na
LT2|1940|ST19|na|na
Malaysia-1|2004|ST19|Homo_sapiens|Blood
Cluster 1
COL_020|2016|SD|Homo_sapiens|Hemoculture
COL_007|2017|ST7478|Homo_sapiens|Urine
COL_010|2015|ST7478|Homo_sapiens|Hemoculture
COL_036|2021|na|Homo_sapiens|na
COL_045|2021|na|Homo_sapiens|na
COL_011|2015|ST19|Homo_sapiens|Hemoculture
COL_012|2015|ST19|Homo_sapiens|Hemoculture
COL_024|2018|na|Homo_sapiens|na
COL_019|2016|SD|Homo_sapiens|Hemoculture
COL_003|2016|ST19|Homo_sapiens|Hemoculture
COL_029|2019|na|Homo_sapiens|na
Pork-402|2020|na|Sus_scrofa|na
Pork-403|2020|na|Sus_scrofa|na
COL_023|2016|ST19|Homo_sapiens|Hemoculture
COL_055|2019|na|Homo_sapiens|na
COL_028|2018|na|Homo_sapiens|na
COL_056|2019|na|Homo_sapiens|na
COL_006|2017|ST19|Homo_sapiens|Stool
Pork-123|2020|na|Sus_scrofa|na
COL_034|2021|na|Homo_sapiens|na
COL_031|2020|na|Homo_sapiens|na
COL_032|2020|na|Homo_sapiens|na
COL_038|2022|na|Homo_sapiens|na
Pork-1777|2020|na|Sus_scrofa|na
Pork-67A|2020|na|Sus_scrofa|na
Pork-35b|2021|na|Sus_scrofa|na
Pork-23b|2021|na|Sus_scrofa|na
Pork-19c|2021|na|Sus_scrofa|na
Pork-20d|2021|na|Sus_scrofa|na
Pork-1b|2021|na|Sus_scrofa|na
Pork-12a|2021|na|Sus_scrofa|na
Pork-24c|2021|na|Sus_scrofa|na
Pork-26c|2021|na|Sus_scrofa|na
Pork-17|2021|na|Sus_scrofa|na
Pork-60|2021|na|Sus_scrofa|na
COL_017|2016|ST19|Homo_sapiens|Hemoculture
COL_026|2019|na|Homo_sapiens|na
COL_040|2021|na|Homo_sapiens|na
Pork-896|2020|na|Sus_scrofa|na
COL_013|2015|ST19|Homo_sapiens|Hemoculture
Pork-286|2020|na|Sus_scrofa|na
Pork-1916|2020|na|Sus_scrofa|na
Pork-218|2020|na|Sus_scrofa|na
COL_054|2019|na|Homo_sapiens|na
Pork-1641|2020|na|Sus_scrofa|na
Pork-10b|2020|na|Sus_scrofa|na
Pork-105|2020|na|Sus_scrofa|na
Pork-100d|2021|na|Sus_scrofa|na
Pork-103c|2021|na|Sus_scrofa|na
COL_043|2021|na|Homo_sapiens|na
COL_047|2022|na|Homo_sapiens|na
COL_042|2021|na|Homo_sapiens|na
Pork-102|2020|na|Sus_scrofa|na
Pork-26|2020|na|Sus_scrofa|na
Pork-6|2020|na|Sus_scrofa|na
Pork-129c|2021|na|Sus_scrofa|na
Pork-152b|2021|na|Sus_scrofa|na
Pork-150a|2021|na|Sus_scrofa|na
Pork-165a|2021|na|Sus_scrofa|na
Cluster 4
SPAIN-LSP-389-97|1997|ST19|Homo_sapiens|Stool
Spain-3|211|ST19|Bos_taurus|na
Canada_16|2016|na|Homo_sapiens|Stool
Canada_6|2010|na|Homo_sapiens|Stool
Canada-Pork_18|2008|na|Sus_scrofa|na
Canada_13|2010|ST19|Homo_sapiens|Stool
Canada_9|2011|na|Homo_sapiens|Stool
Canada_14|2010|ST19|Homo_sapiens|na
Canada_15|2010|ST19|Homo_sapiens|Stool
Cluster 5
Canada_1|2008|na|Homo_sapiens|Stool
Canada_19|2013|na|Bos_taurus|na
Canada_31|2012|ST19|Homo_sapiens|Stool
Canada_25|2010|ST19|Homo_sapiens|Stool
USA-17|2010|ST19|Homo_sapiens|na
COL_021|2016|ST19|Homo_sapiens|Hemoculture
USA-7|2006|ST19|Food|na
Canada_24|2009|ST19|Homo_sapiens|Stool
USA-4|2010|ST19|Homo_sapiens|Stool
Denmark-10|2016|na|Homo_sapiens|na
USA-16|2011|ST19|Food|na
Canada_7|2008|ST19|Homo_sapiens|Stool
Canada_10|2009|ST19|Homo_sapiens|Stool
Canada_5|2008|ST19|Homo_sapiens|Stool
USA-11|2011|ST19|Food|na
USA-8|2015|ST19|Food|na
USA-12|2015|ST19|Food|na
Canada_12|2010|ST19|Homo_sapiens|Stool
USA-10|2007|ST19|Food|na
USA-9|2015|ST19|Food|na
Canada_22|2011|ST19|Homo_sapiens|Stool
USA-13|2006|ST19|Food|na
Canada_20|2010|ST19|Homo_sapiens|Stool
USA-15|2010|ST19|Food|na
Canada_23|2010|ST19|Homo_sapiens|Stool
Canada_29|2011|ST34|Homo_sapiens|Stool
Canada_2|2008|ST19|Homo_sapiens|Stool
USA-14|2010|ST19|Food|na
USA-5|2015|ST34|Homo_sapiens|Stool
Japan_1|2007|ST34|Homo_sapiens|Stool
Japan_3|1998|ST34|Sus_scrofa|na
Denmark-14|2009|na|Homo_sapiens|na
Denmark-15|2006|na|Homo_sapiens|na
Japan_4|2014|ST34|Bos_taurus|na
Canada_28|2010|ST34|Homo_sapiens|Stool
Denmark-2|2017|na|Homo_sapiens|na
New-Zealand-1|2020|na|Homo_sapiens|na
Cluster 3
Denmark-13|2017|na|Homo_sapiens|na
Denmark-6|2015|na|Homo_sapiens|na
Spain-1|2007|ST34|Homo_sapiens|Stool
Denmark-5|2015|na|Homo_sapiens|na
Canada_33|2012|ST34|Homo_sapiens|Stool
Denmark-12|2017|na|Homo_sapiens|na
Canada_8|2009|ST34|Homo_sapiens|Stool
Canada_11|2010|ST34|Homo_sapiens|Stool
Canada_26|2009|ST34|Homo_sapiens|Stool
Canada_3|2008|na|Homo_sapiens|Stool
COL_002|2015|ST34|Homo_sapiens|Stool
Denmark-8|2015|na|Homo_sapiens|na
Denmark-4|2012|na|Homo_sapiens|na
Denmark-7|2015|na|Homo_sapiens|na
Japan_6|2014|ST34|Bos_taurus|na
Japan_5|2014|ST34|Bos_taurus|na
Japan_7|2015|ST34|Bos_taurus|na
New-Zealand-2|2021|na|Homo_sapiens|na
Portugal-6|2012|na|Homo_sapiens|Stool
Portugal-7|2018|na|Homo_sapiens|Blood
Canada_32|2012|ST34|Homo_sapiens|Stool
Japan_2|2008|ST34|Bos_taurus|na
New-Zealand-3|2021|na|Homo_sapiens|na
USA-3|2014|na|Homo_sapiens|Stool
Canada_36|2014|ST2379|Homo_sapiens|Stool
USA-18|2010|ST2379|Homo_sapiens|na
USA-2|2016|na|Homo_sapiens|Stool
Canada_38|2016|ST34|Homo_sapiens|Stool
COL_033|2021|na|Homo_sapiens|na
USA-1|2016|na|Homo_sapiens|Stool
Australia_6|2014|ST34|Homo_sapiens|Stool
Canada_4|2008|ST34|Homo_sapiens|Stool
Canada_21|2016|ST34|Homo_sapiens|Stool
Denmark-1|2018|na|Homo_sapiens|na
Canada_27|2012|ST34|Homo_sapiens|Stool
Australia_1|2020|ST34|Homo_sapiens|Stool
Australia_2|2020|ST34|Homo_sapiens|Urine
Australia_3|2020|ST34|Homo_sapiens|Blood
Canada_37|2015|ST34|Homo_sapiens|Stool
Denmark-16|2018|na|Homo_sapiens|na
Ireland-1|2016|na|Homo_sapiens|na
UK-EUROPE-SO4698-09|2009|ST34|Bos_taurus|na
Australia-Pork_7|2017|ST34|Sus_scrofa|na
Australia_4|2018|ST34|Homo_sapiens|Stool
Australia_5|2016|ST34|Homo_sapiens|Stool
Portugal-5|2010|na|Homo_sapiens|Stool
Canada_35|2014|ST34|Homo_sapiens|Stool
COL_058|2019|na|Homo_sapiens|na
COL_057|2019|na|Homo_sapiens|na
COL_059|2019|na|Homo_sapiens|na
COL_030|2019|na|Homo_sapiens|na
Pork-261|2020|na|Sus_scrofa|na
COL_052|2019|na|Homo_sapiens|na
Pork-463|2020|na|Sus_scrofa|na
COL_014|2015|ST34|Homo_sapiens|Hemoculture
COL_015|2016|ST34|Homo_sapiens|Hemoculture
COL_025|2020|na|Homo_sapiens|na
Pork-500|2020|na|Sus_scrofa|na
Portugal-1|2019|na|Homo_sapiens|na
Portugal-2|2019|na|Homo_sapiens|na
Portugal-3|2019|na|Homo_sapiens|na
1775
1800
1825
1850
1875
1900
1925
1950
1975
2000
2025
2050
2075
