# Supplementary material for: Comparative genomics of the monophasic variant of Salmonella Typhimurium: analysis of Colombian genomes and their relationship with international lineages
Source: Microbiol Spectr. 2026 Sep 17;14(10):e03870-25. doi: 10.1128/spectrum.03870-25 (PMC13689331; doi:10.1128/spectrum.03870-25)
Supplement: Supplementary file 2 [file spectrum.03870-25-s0002.pdf]

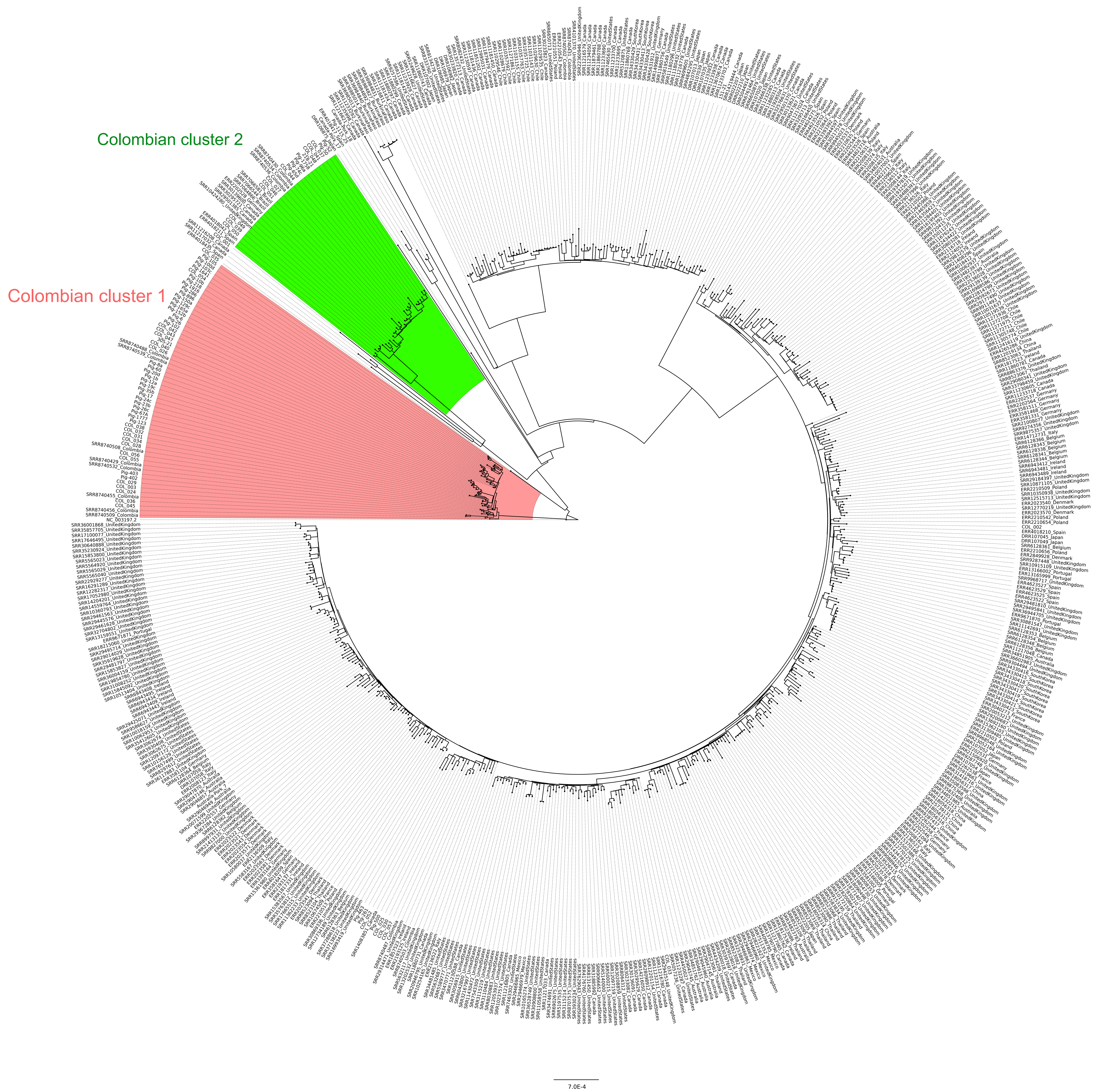
Colombian cluster 2
Colombian cluster 1
7.0E-4

Supplementary Figure 2. Global phylogenetic context of Colombian *Salmonella* Typhimurium monophasic variant (STVM) genomes retrieved from Enterobase.

Maximum-likelihood phylogenetic tree constructed from core-genome SNP alignments of 602 STVM genomes (plus the *S.* Typhimurium LT2 reference genome NC_003197.2 as outgroup), including 95 Colombian isolates (human and swine sources, 2015–2022) and 507 international genomes spanning multiple continents. The tree was used to establish the global phylogenetic context of the five lineages identified in the main analysis. Two major Colombian endemic clades are highlighted: Colombian cluster 1 (pink shading; *n* = 62, predominantly ST19, Colombian Lineage 1) and Colombian cluster 2 (green shading; *n* = 22, ST19, Colombian Lineage 2). The interactive version of this tree is available at: https://microreact.org/project/5zJKTrm94gQTkDGiMvGoQp-newenterobasetree
