# Supplementary material for: Comparative genomics of the monophasic variant of Salmonella Typhimurium: analysis of Colombian genomes and their relationship with international lineages
Source: Microbiol Spectr. 2026 Sep 17;14(10):e03870-25. doi: 10.1128/spectrum.03870-25 (PMC13689331; doi:10.1128/spectrum.03870-25)
Supplement: Supplementary file 3 [file spectrum.03870-25-s0003.docx]

**Supplementary Figures Legends.**

**Supplementary Figure 1.Time phylogeny analysis of Colombian vs. international STVM isolates.** Time-resolved phylogeny of Colombian and international STVM isolates. Maximum-likelihood phylogeny (selected by Bayesian Information Criterion, BIC) showing the phylodynamics of STVM samples and the estimated divergence dates; the topology reveals two Colombian lineages (highlighted in purple and green) with clonal expansion dated to 2003–2005, while the international clones show estimated emergence dates in the 1990s, consistent with previous reports.

**Supplementary Figure 2. Global phylogenetic context of Colombian *Salmonella* Typhimurium monophasic variant (STVM) genomes retrieved from Enterobase.**

Maximum-likelihood phylogenetic tree constructed from core-genome SNP alignments of 602 STVM genomes (plus the *S.* Typhimurium LT2 reference genome NC_003197.2 as outgroup), including 95 Colombian isolates (human and swine sources, 2015–2022) and 507 international genomes spanning multiple continents. The tree was used to establish the global phylogenetic context of the five lineages identified in the main analysis. Two major Colombian endemic clades are highlighted: Colombian cluster 1 (pink shading; *n* = 62, predominantly ST19, Colombian Lineage 1) and Colombian cluster 2 (green shading; *n* = 22, ST19, Colombian Lineage 2). The interactive version of this tree is available at: https://microreact.org/project/5zJKTrm94gQTkDGiMvGoQp-newenterobasetree
